# Sparse-View Ultrasound Diffraction Tomography Using Compressed Sensing with Nonuniform FFT

**DOI:** 10.1155/2014/329350

**Published:** 2014-04-24

**Authors:** Shaoyan Hua, Mingyue Ding, Ming Yuchi

**Affiliations:** Image Processing and Intelligence Control Key Laboratory of Education Ministry of China, Department of Biomedical Engineering, School of Life Science and Technology, Huazhong University of Science and Technology, Wuhan 430074, China

## Abstract

Accurate reconstruction of the object from sparse-view sampling data is an appealing issue for ultrasound diffraction tomography (UDT). In this paper, we present a reconstruction method based on compressed sensing framework for sparse-view UDT. Due to the piecewise uniform characteristics of anatomy structures, the total variation is introduced into the cost function to find a more faithful sparse representation of the object. The inverse problem of UDT is iteratively resolved by conjugate gradient with nonuniform fast Fourier transform. Simulation results show the effectiveness of the proposed method that the main characteristics of the object can be properly presented with only 16 views. Compared to interpolation and multiband method, the proposed method can provide higher resolution and lower artifacts with the same view number. The robustness to noise and the computation complexity are also discussed.

## 1. Introduction

In recent years, ultrasound diffraction tomography (UDT) has drawn more and more attention in medical imaging field. Different from traditional B-mode ultrasound technique which displays the strength of the echoes with gray scale to show anatomic structure, UDT infers the distribution of acoustic properties such as refractivity, attenuation, and density. Since these acoustic properties of normal and diseased tissues have different value ranges [1], UDT has the potential for providing functional information of the object. For example, in the breast cancer exam, the malignant tumor, the benign mass, and the normal tissue can be differentiated by UDT [2].

Under the assumption of weak scatting, the Fourier diffraction theory (FDT) [3, 4] is adopted for the image reconstruction of UDT. Firstly, the object is illuminated by plane sound wave from one certain direction and the scattering waves are measured and sampled. Secondly, the spatial Fourier transform is performed on the stored data. Under the Born or Rytov approximation [5], the corresponding frequency values are considered to represent the 2D Fourier transform of the object nonuniform distributed along a semicircle arc. The above processes are implemented oriented at various angles around the object to acquire sufficient spatial frequency information. Finally, the UDT image is reconstructed through inverse spatial Fourier transform. To avoid undersampling, normally more spatial frequency samples are required [6]; thus more views from different directions are needed. However, the scan and process time and the associated cost will increase. Besides that, it will impose rigorous requirements on controlling precision of the imaging system. Moreover, redundant information is introduced by multiple views, which results in the waste of the system resources. Hence, accurate reconstruction from nonuniform distributed frequency samples in sparse-view situation has great practical significance.

Currently, in addition to reconstructing the underlying object through beamforming [7], there are other two main approaches based on FDT to recover the complete information from the sparse-view data: interpolation method and iterative method. The former calculates the frequency values on the rectangular grids by a predetermined interpolation function based on sampled frequency on the circular arc grid [8, 9]. Although the interpolation method is computationally efficient, it is liable to introduce coordinate conversion error, which can severely introduce artifacts and distort the image, especially in sparse-view situation. The latter repeatedly corrects the reconstructed image by minimizing the inconsistency between the sampled and the estimated frequency. Through designing appropriate optimization strategy, this process can maximally reduce the reconstruction error and suppress the artifacts. Various researches have been done on the UDT image reconstruction with the iterative method under sparse-view situation. Bronstein et al. [10] proposed an iterative reconstruction framework based on nonuniform fast Fourier transform (NUFFT) and reduced the number of view with broadband sound wave. LaRoque et al. [11] introduced a kind of diffraction tomography with few-view (sparse-view) and limited-angle data through total variation (TV) minimization algorithm for absorberless media. Tingting Li et al. [12] combined TV regularization with the iterative next-neighbor regridding (INNG) algorithm to suppress artifacts and noise.

Compressed sensing (CS) [13, 14] is built on the sparse nature of real signals in certain transform domain. CS has the ability to reconstruct signals with samples which are much less than those required by the Nyquist criterion. CS brings great innovations in image reconstruction and has been widely used in medical imaging field such as MR [15], CT [16, 17], PET [18], and photoacoustic imaging [19, 20]. In recent years, some progress has been made in applying this emerging theory in ultrasound imaging field to reduce the amount of the data and the complexity of the imaging system. Schiffner et al. [21–23] investigated the performance of CS in solving the inverse scattering problem in pule-echo diagnostic ultrasound imaging under the constraint that the scatterer distribution is sparse. With the same assumption, Wagner et al. [24–26] proposed a method based on finite rate of innovation and Xampling for the reconstruction of the beamformed image from channel RF data. Shen et al. [27, 28] presented a measurement-domain adaptive beamforming approach based on distributed CS to reconstruct an image of sparse targets. Achim et al. [29] introduced a framework based on CS for ultrasonic signals reconstruction under the assumption of RF echoes with *α*-stable distributions. Liebgott et al. [30–32] studied the feasibility of CS for the reconstruction of channel RF data, the quantity of channel RF data is reduced through introducing the wave atoms as a representation basis for prebeamformed RF signal. Quinsac et al. [33–37] and Dobigeon et al. [38] applied CS theory to recover beamformed 2D RF images using conjugate gradient descent or Bayesian approach. Zobly et al. [39, 40] and Richy et al. [41] applied CS for doppler imaging.

In this paper, we propose an iterative reconstruction method based on CS for sparse-view UDT. The above researchers mainly focused on applying CS to the pulse-echo imaging systems. To the best of our knowledge, CS was rarely applied in the investigation of UDT image reconstruction, and this is the main motivation of this work. According to FDT, the sample points are bounded in a circle with the radius of $\sqrt{2}k_{0}$ (*k*_0_: wave number) [9] for transmission UDT; this results in a sharp sampling cutoff in spatial frequency space. Thus, the reconstructed object is a low-pass version of the original and the quality of image will be distorted by the Gibbs aliasing. This problem can be further deteriorated in the sparse-view situation due to limited sample data. In this context, besides the general *l*_1_ norm constraint, we introduce TV penalty into the cost function, which can reduce the oscillation and preserve edges of the object [42]. Empirical observations [10] showed that the majority of nature images, particularly medical images, demonstrated piecewise continuous behavior; that is, parts of anatomy structures were supposed to show uniform characteristics, which belonged to the class of functions of bounded TV [43]. Furthermore, to improve computational efficiency, a fast and accurate NUFFT is adopted to calculate the forward scatter field.

Through the numerical simulation, the proposed method is verified and compared with interpolation method and iteration method with broadband signal [10]. The quantitative evaluation and the robustness to noise of the method are discussed. Simulation shows that the object can be faithfully reconstructed in sparse-view situation without noticeable loss of image quality and the reconstructed error is reduced.

The rest of the paper is organized as follows. The basic principle of UDT and CS theory are described in Section 2. The proposed method is presented in Section 3.  Section 4 describes the simulation experiments for UDT image reconstruction and results.  Section 5 provides discussion about noise robustness and computational complexity of the proposed method. Conclusions and future work are summarized in Section 6.

## 2. Background

### 2.1. UDT Based on FDT

**Figure 1:**
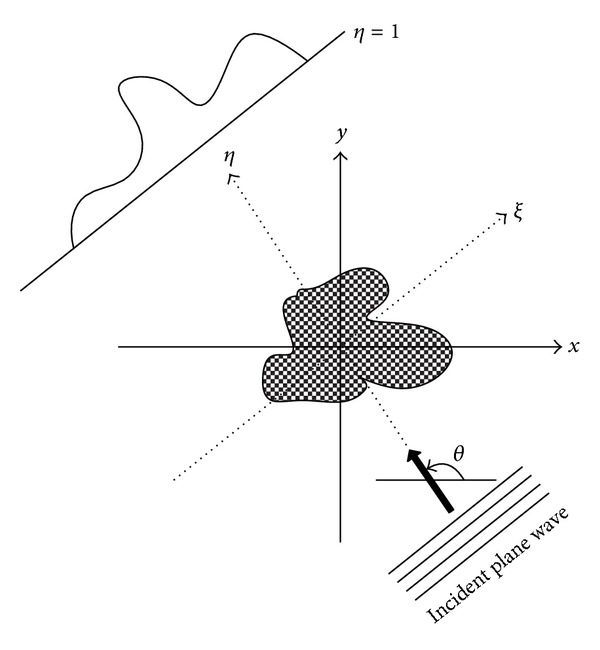
Scheme of 2D ultrasound diffraction tomography: illuminating the object with plane wave at an angle *θ* and the scattered measured along *η* = *l*.

The classical 2D UDT imaging configuration is shown in Figure 1. The inhomogeneous object with a distribution function *f*(**r**) = *n* ^2^(**r**) − 1, **r** = (*x*, *y*), is surrounded by homogeneous medium such as water; the *n*(**r**) is refractive index. The reconstruction object of UDT is to infer the unknown *f*(**r**) through transmitted signals measured by ultrasound transducer.

Assume the object is illuminated by a monochromatic plane wave with wave number *k*_0_ and angular frequency *ω*_0_ at an angle *θ*. The wave is scattered within and at the boundary of the inhomogeneous object. Set up a rotated cartesian coordinate system (*ξ*, *η*) such that *η*-axis is coincident with the view angle as shown in Figure 1. The relation between (*x*, *y*) and (*ξ*, *η*) can be derived through coordinate transformation: *ξ* = cos⁡(*θ*)*x* + sin(*θ*)*y*, *η* = −sin(*θ*)*x* + cos⁡(*θ*)*y*.

The sound pressure field *u*(**r**) satisfies the following wave equation [4]:

(1) $$\begin{array}{c} \left(\nabla^{2}+k_{0}^{2}\right)u\left(r\right)=-k_{0}^{2}f\left(r\right)u\left(r\right), \end{array}$$

where ∇^2^ denotes the Laplacian operator. *u*(**r**) can be modeled as the superposition of the incident wave *u*_*i*,*θ*_(**r**) and the scatter wave *u*_*s*,*θ*_(**r**): *u*(**r**) = *u*_*i*,*θ*_(**r**) + *u*_*s*,*θ*_(**r**).

**Figure 2:**
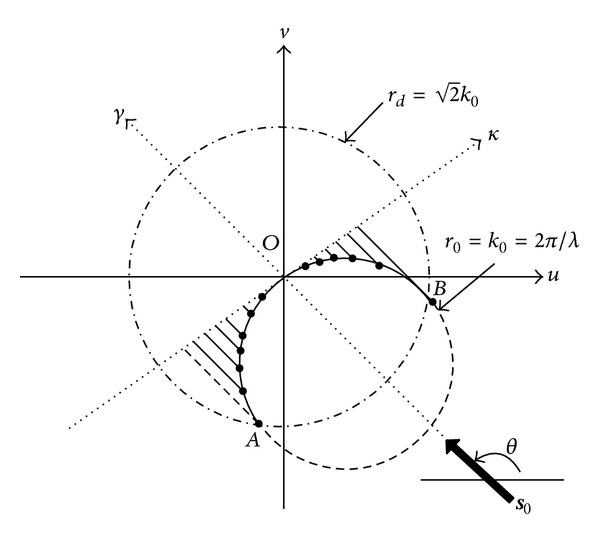
Fourier diffraction projection theorem: the Fourier transform of the scattered data equals the Fourier transform of the object along semicircle AOB.

Under the assumption of weak scattering, *u*_*s*,*θ*_(**r**) ≪ *u*_*i*,*θ*_(**r**), or first order Born, or Rytov approximation, one can derive the so-called FDT which relates the scattered field measured along the line *η* = *l* to the object by Fourier transform:

(2) $$\begin{array}{c} U_{s,\theta }\left(\kappa \right)=\frac{k_{0}^{2}U_{0}}{2jy}e^{jyl}\iint_{-\infty }^{+\infty }f\left(r\right)e^{-j[\kappa \xi +(\gamma -k_{0})\eta ]}dr, \end{array}$$

where *U*_*s*,*θ*_(*κ*) is the Fourier transform of received scattered data *u*_*s*,*θ*_(*ξ*) with respect to *ξ*:

(3) $$\begin{array}{c} U_{s,\theta }\left(\kappa \right)=\overset{+\infty }{\underset{-\infty }{\int }}u_{s,\theta }\left(\xi \right)e^{-j\kappa \xi }d\xi \end{array}$$

and $y=\sqrt{k_{0}^{2}-\kappa^{2}}$, |*κ*| ≤ *k*_0_, and *U*_0_ is the complex amplitude of the illuminating plane wave. The quantity of *U*_*s*,*θ*_(*κ*) in (2) is known or measurable, and the integral *Q*_*θ*_(*κ*) = ∬_−*∞*_^+*∞*^ *f*(**r**)*e* ^−*j*[*κξ*+(*γ*−*k*_0_)*η*]^ *d* **r** contains the underlying object *f*(**r**) that we want to reconstruct. Detailed analysis reveals that *Q*_*θ*_(*κ*) is the Fourier transform of the object along a semicircle of radius *k*_0_ and centered at −*k*_0_ **s**_0_ [4], as the arc AOB depicted in Figure 2, and **s**_0_ is the unit vector along direction *θ*. Mathematically, the image reconstruction by (2) is a typical inverse problem which can be expressed as the following formulation:

(4) $$\begin{array}{c} F=\Phi \left(f\right), \end{array}$$

where *F* is the sample data in *k*-space, Φ is the diffraction operator, and *f* is the object *f*(**r**) which we want to reconstruct. For formulation (4), classical interpolation method based on FDT is not applicable in sparse-view situation due to violating Nyquist limitation. With fast and accurate nonuniform fast Fourier transform, we propose a CS framework for UDT reconstruction in sparse-view situation to improve the image quality of the reconstruction.

### 2.2. Compressed Sensing: A Short Overview

Compressed sensing is a kind of signal processing technique for efficiently acquiring and reconstructing a signal. CS has been increasingly adopted in a variety of applications by applied mathematicians, computer scientists, and engineers since it was initiated in 2006 [44]. The idea of CS can be expressed by the following linear measurement model:

(5) $$\begin{array}{c} y=\Phi x, \end{array}$$

where **x** ∈ ℝ^*n*^ is the unknown signal such as an image that we want to reconstruct, **y** is the measured signal, and Φ is one *m* × *n*  (*m* < *n*) measure matrix, which is decided by the imaging system. In this work, Φ is the Fourier transform operator and **y** is the corresponding Fourier transform of the received scatter field. Assume the unknown signal **x** can be sparsely represented in terms of a known basis:

(6) $$\begin{array}{c} x=\Psi s, \end{array}$$

where Ψ is the basis, **s** is the corresponding representation coefficients. Here, the sparse means that the number of nonzero coefficients of **s** is small.

Substituting (6) into (5), we obtain (7) as follows:

(7) $$\begin{array}{c} y=\Theta s, \end{array}$$

where Θ = ΦΨ; since *m* < *n*, the matrix Θ is not invertible. Taking advantage of the sparsity of **s**, CS theory shows that if the Θ meets the so-called restricted isometry property (RIP) condition, we can exactly recover **s** with overwhelming probability by solving the following minimization problem [45, 46]:

(8) $$\begin{array}{c} \underset{s}{\min}\left\{{||s||}_{0}:\Theta s=y\right\}, \end{array}$$

where ||·||_0_ norm counts the number of no-zero entry of a vector. Equation (8) seeks the sparsest one among all the possible solutions of **y** = Θ**s**.

However, (8) belongs to the class of NP-hard problem, which is difficult to obtain solutions for nearly all real applications. One computationally tractable alternative for (8) is to solve the following *l*_1_ problem [13, 45]:

(9) $$\begin{array}{c} \underset{s}{\min}\left\{{||s||}_{1}:\Theta s=y\right\}, \end{array}$$

where ||**s**||_1_ = ∑|*s*_*i*_|.

The above CS theory requires **x** to be sparse and **y** = Φ**x** exactly, whereas, in most practical situations, the object is approximately sparse or compressible. Here, approximately sparse means that **x** contains a small number of components with magnitudes significantly larger than those of the rest, which are not necessarily zero; compressible means the coefficients of **s** decay exponentially in absolute value. To address these problems, Candès et al. [47] extended (9) to the following form:

(10) $$\begin{array}{c} \underset{s}{\min}\left\{{||s||}_{1}:{||\Theta s-y||}_{2}^{2}⩽\sigma^{2}\right\}, \end{array}$$

where ||**s**||_2_ = (∑|*s*_*i*_|^2^)^1/2^ and *σ* ^2^ represents energy bound of error.

Considering that (10) is a convex optimization problem, it can be further recast as the following regularization equation [48]:

(11) $$\begin{array}{c} \underset{s}{\min}\left\{\alpha {||s||}_{1}+{||\Theta s-y||}_{2}^{2}\right\}, \end{array}$$

where *α* is a regularization parameter.

## 3. Compressed Sensing for UDT Imaging Reconstruction

**Figure 3:**
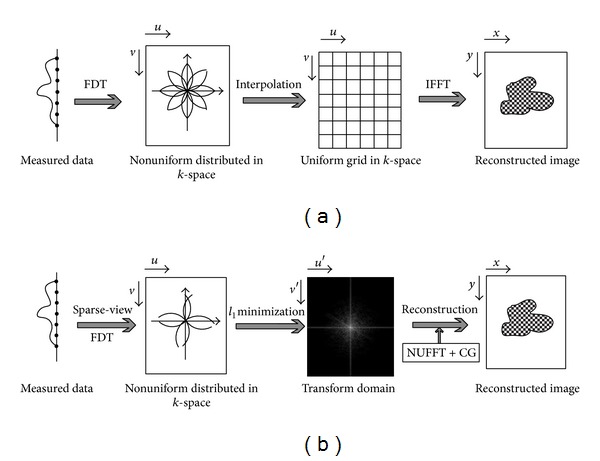
Schematic of the frequency interpolation method (a) and proposed method (b). In (a), the object is reconstructed through IFFT after frequency interpolation. In (b), the object is reconstructed through CG and NUFFT.

In this section, we present an image reconstruction method for UDT in 2D case, while it can be readily extended to 3D.  Figure 3 provides the comparison between the interpolation method and the proposed scheme, which is composed of three major steps. Firstly, the object is illuminated from random angles and the number of views can be far below that restricted by the Nyquist limitation. The measured sparse-view data are processed by Fourier transform to obtain the corresponding spatial frequency samples along semicircular arcs oriented at the view angles. Secondly, the inverse problem (4) is formulated within CS framework by building the measure matrix Φ and exploiting the sparsity of the object. Thirdly, the object is reconstructed through the NUFFT and CG algorithm.

### 3.1. Sparse-View Data Sampling

As we discussed in Section 2.2, when the sensing matrix Θ satisfies the RIP [45, 46], the NP-hard inverse problem (8) can be transformed to computationally tractable *l*_1_ norm minimization problem ((9) or (11)). However, even for moderate dimensional operators Θ, it is computationally impractical to verify the RIP. Fortunately, a few classes of matrices are shown to hold RIP for almost certainly. It is shown in [49, 50] that, when Φ is a Gaussian or partial Fourier, that is, the entries of Φ are randomly selected using a Gaussian pdf or *m* rows of Φ are randomly selected from the rows of *n* × *n* Fourier matrix, Θ satisfies RIP.

For UDT scanner, the sample operator coincides with the matrix mentioned above since the samples in spatial frequency domain are obtained through FDT, Which makes it possible to reconstruct the object through *l*_1_ minimization with sparse data. Here, we generate the sparse-view data by randomly choosing view angles and the number of view is much less than that required by the conventional interpolation method.

### 3.2. Inverse Problem Formulation under CS Framework

The sparse-view data sampling in Section 3.1 generates the measured value *y* in problem (9). To formulate the UDT inverse problem under CS framework, the sensing matrix Θ = ΦΨ in (9) must be constructed. Here, Φ is dependent on the imaging principle of UDT, while Ψ is the basis for the object to be reconstructed. The explicit expression of Φ and Ψ for UDT will be derived as below.

Since the object has limited physical dimensions, we assume the object *f*(**r**) or *f*(*x*, *y*) in cartesian coordinate system has bounded support [−*C*, *C*]×[−*C*, *C*]; that is, *f*(*x*, *y*) = 0 when |*x*| > *C* or |*y*| > *C*. Let *f*_*d*_ ∈ ℝ^(*n*_*d*_+1)×(*n*_*d*_+1)^, (*n*_*d*_ = 2⌈*C*/*T*⌉) be the discrete form of the underlying object that we want to reconstruct, where *T* is the sample period for *x*- and *y*-axis. *F*(*u*, *v*) and *F*_*d*_(*u*, *v*) are the Fourier transform of *f*(*x*, *y*) and *f*_*d*_(*n*_1_, *n*_2_), respectively. Assume the frequency response of *f*(*x*, *y*) is band-limited; that is, there exists a cutoff frequency *W* such that |*F*(*u*, *v*)| ≈ 0, when |*u*| > *W* or |*v*| > *W*. In practice, the loss of resolution by this band limit is negligible; the reconstructed imaging quantity is more influenced by other factors such as the aperture sizes of transmitting and receiving transducers [9]. Based on the Nyquist theorem, if *T* < 1/2*W*, *F*_*d*_(*u*, *v*) ≈ *F*(*u*, *v*) for |*u*| < *W* and |*v*| < *W*.

Since the receiving array of UDT has a limited number of elements, we denote the measured discrete field ${\hat{u}}_{s,\theta }=u_{s,\theta }(n\tau )$, where *n* is the number of elements and *τ* is pitch (the distance between the centers of two adjacent elements). *τ* is the spatial sampling interval of UDT system. ${\hat{U}}_{s,\theta }(\kappa )$ and *U*_*s*,*θ*_(*κ*) are the Fourier transform of ${\hat{u}}_{s,\theta }$ and *u*_*s*,*θ*_(*ξ*), respectively. ${\hat{U}}_{s,\theta }(\kappa )\to U_{s,\theta }(\kappa )$ for all *κ* ∈ ℝ as *τ* → 0. The relation between (*u*, *v*) and (*κ*, *γ*) can be formulated as the following equations:

(12) $$\begin{array}{c} \kappa =𝒰\left(u,v\right),\\ \gamma =𝒱\left(u,v\right), \end{array}$$

where *𝒰*, *𝒱* are derived from the coordinate transform and (2); let arc grid points (*κ*_1_, *γ*_1_) and (*κ*_2_, *γ*_2_) (−*k*_0_ ≤ *κ*_1_ ≤ 0,  0 ≤ *κ*_2_ ≤ *k*_0_) be on half arc* AO*,* OB*, respectively; then we can get $\kappa_{i}={\left(-1\right)}^{i}k_{0}\sin(2\;\arcsin(\sqrt{u^{2}+v^{2}}/2k_{0}))$, $\gamma_{i}=\sqrt{k_{0}^{2}-\kappa_{i}^{2}}$, *i* = 1, 2. For more details about this transformation please refer to [4].

According to (2) and (12), if ${\hat{U}}_{s,\theta }(\kappa )$ sufficiently approximates *U*_*s*,*θ*_(*κ*) and |*u*| < *W*, |*v*| < *W*, then we have ${\hat{U}}_{s,\theta }(\kappa )\approx U_{s,\theta }(\kappa )=F(\kappa ,\gamma )=F(u,v)\approx F_{d}(u,v)$. Generally, this requirement can be satisfied in the real UDT system because the pitch of normal medical ultrasound probe is among 0.5*λ* ~ 2*λ* (*λ* is the sound wave length).

Let *Ω*_1_ = {*θ*_1_, *θ*_2_,…, *θ*_*N*_1__} be the finite set of view angles. In UDT system, *U*_*s*,*θ*_(*κ*) is obtained over *Ω*_1_. Then the corresponding ${\hat{U}}_{s,\theta }(\kappa )$ can be calculated offline for all *κ* ∈ *Ω*_2_ = {*κ*_1_, *κ*_2_,…, *κ*_*N*_2__} (3). Therefore, the *f*_*d*_ can be reconstructed through the set of observation ${\hat{U}}_{s,\theta }(\kappa )|(\theta ,\kappa )\in (\Omega_{1},\Omega_{2})$:

(13) (Tκ0)2U02jγejγl∑n1=⌊−C/T⌋⌈C/T⌉ ∑n2=⌊−C/T⌋⌈C/T⌉fdn1,n2e−2πj(n1u+n2v)T =U^s,θ(κ)+ns,θ(κ).

This set of equations can be written in matrix form: Φ*f*_*d*_ = *F* + *n*, where *Ω* = (*Ω*_1_, *Ω*_2_), Φ ∈ *ℂ* ^|*Ω*|×(*n*_*d*_ + 1)^2^^, *f*_*d*_ ∈ ℝ^(*n*_*d*_ + 1)^2^^, −*n*_*d*_/2 ≤ *n*_1_, *n*_2_ ≤ *n*_*d*_/2, *F*, *n* ∈ *ℂ* ^|*Ω*|^, *n*_*s*,*θ*_(*κ*), or *n* is approximation error. With (13), Φ in inverse problem (9) is obtained.

CS theory utilizes the sparse nature of the object and reconstructs the object by minimizing the corresponding *l*_1_ norm in transform domain. According to FDT, the spatial frequency samples are distributed along the arc AOB (Figure 2). As the incident wave revolves around the object, the AOB describes a disk of radius $\sqrt{2}k_{0}$ centered at the origin; that is, the reconstructed object is a low-pass version of the original. Besides that, we also exploit the fact that the structural morphology of human soft tissue is expected to demonstrate piecewise continuous behavior. That means the object belongs to the class of bounded TV [43] and the gradient of the object is sparse. In this work, the sparsity of the underlying object is exploited not only through wavelet transform which provides sparse representations for rapidly varied regions, but also by TV which affords sparse transformation for piecewise smooth object. Furthermore, TV constraint can help to suppress Gibbs effect and preserve edges [10].

For a discrete object *f*_*d*_, TV is defined as

(14) $$\begin{array}{c} \text{TV}\left(f_{d}\right)=\underset{n_{1},n_{2}}{\sum }\sqrt{{\left(D_{n_{1},n_{2}}^{h}f_{d}\right)}^{2}+{\left(D_{n_{1},n_{2}}^{v}f_{d}\right)}^{2}}=\underset{n_{1},n_{2}}{\sum }{||D_{n_{1}n_{2}}f_{d}||}_{2}, \end{array}$$

where *D*_*n*_1_,*n*_2__^*h*^, *D*_*n*_1_,*n*_2__^*v*^ denote the forward finite difference operator in horizontal (*h*) and vertical (*v*) coordinates, respectively. Combining *l*_1_ norm with TV constraints, we extend the problem (11) to the following minimization problem:

(15) $$\begin{array}{c} \underset{s}{\min}\;G\left(s\right)=\alpha \text{TV}\left(\Psi s\right)+\beta {||s||}_{1}+{||F-\Phi \Psi s||}_{2}^{2}, \end{array}$$

where *α*, *β* are two positive regularization parameters, *F* is spatial frequency samples, Ψ is the selected basis for sparse representation of the object *f*_*d*_, and **s** is the coefficients of *f*_*d*_ in basis Ψ.

### 3.3. Object Reconstruction

An iterative method based on CG is adopted to solve the inverse problem (15). For UDT system, the receive elements are equally spaced that means the *u*_*s*,*θ*_(*ξ*) along *η* = *l* is equally spaced sampled. However, the measurements of *Q*_*θ*_(*κ*) are unequally spaced distributed, since the measurements along the line are projected perpendicularly onto frequency domain of the object along semicircular arc. Thus, the Fourier transform must be computed for every nonuniform frequency points (ΦΨ**s** or Φ*f*_*d*_). Although the result of direct nonuniform discrete Fourier transform (NDFT) is exact, the computation time required by the NDFT restricts its real application. To speed up, a fast NUFFT is employed to approximate NDFT in every iteration of reconstruction.

#### 3.3.1. Conjugate Gradient Method

To solve the inverse problem (15) iteratively by CG, the gradient of the objective function *G*(**s**) must be computed as:

(16) $$\begin{array}{c} \nabla G\left(s\right)=2{\left(\Phi \Psi \right)}^{\prime }\left(\Phi \Psi s-F\right)+\alpha \nabla \left(\text{TV}\left(\Psi s\right)\right)+\beta \nabla \left({||s||}_{1}\right), \end{array}$$

where (·)′ represents the conjugate transpose. Since the absolute value function in *l*_1_ norm and TV is nonsmooth function, we use approximation techniques to compute the corresponding gradient. For *l*_1_ norm, the absolute value function is approximated with a smooth function by using the relation ${||z||}_{1}\approx \sqrt{z^{\prime }z+ɛ}$, where *ɛ* is a small positive smooth parameter. For TV, we use the following approximation strategy to avoid a zero denominator: ${||D_{n_{1}n_{2}}f_{d}||}_{2}\approx \sqrt{{\left(D_{n_{1},n_{2}}^{h}f_{d}\right)}^{2}+{\left(D_{n_{1},n_{2}}^{v}f_{d}\right)}^{2}+\mu }$, where *μ* is a small positive parameter. Therefore the gradient of *l*_1_ norm and TV can be calculated:

(17) $$\begin{array}{c} \nabla \left({||z||}_{1}\right)=\frac{z}{\sqrt{z^{\prime }z+ɛ}}, \end{array}$$

(18) ∇||Dn1,n2fd||2=Dn1,n2hfd||Dn1,n2fd||2+Dn1,n2vfd||Dn1,n2fd||2−Dn1,n2−1hfd||Dn1,n2−1fd||2 −Dn1−1,n2vfd||Dn1−1,n2fd||2.

#### 3.3.2. NUFFT

NUFFT developed by Fessler and Sutton [51] is adopted. Consider the following 1D NUFFT case:

(19) $$\begin{array}{c} F\left(\omega_{m}\right)=\overset{N-1}{\underset{n=0}{\sum }}f_{n}e^{-i\omega_{m}n},\;m=1,\ldots ,M, \end{array}$$

where **f** = (*f*_0_,…, *f*_*N*−1_) is a vector of equally spaced samples of a signal and **ω** = (*ω*_1_,…, *ω*_*M*_) is a vector of nonuniform distributed frequencies. In matrix notation

(20) $$\begin{array}{c} F=\Phi f, \end{array}$$

where Φ ∈ *ℂ* ^*M*×*N*^ : Φ = (*ϕ*_1_,…,*ϕ*_*M*_)^*T*^ is nonuniform Fourier transform matrix. The NUFFT is implemented by two steps: firstly, project **f** on an oversampled uniform Fourier basis Λ ∈ *ℂ* ^*qM*×*N*^ by standard FFT

(21) $$\begin{array}{c} Z=\Lambda f; \end{array}$$

that is,

(22) $$\begin{array}{c} Z_{k}=\overset{N-1}{\underset{n=0}{\sum }}f_{n}e^{-i(2\pi /K)kn},\;k=0,\ldots ,K-1, \end{array}$$

where *K* = *qM*. Secondly, approximate each *F*(*ω*_*m*_) by interpolating the *Z*_*k*_ using *p* uniform samples

(23) $$\begin{array}{c} F\left(\omega_{m}\right)≃\hat{F}\left(\omega_{m}\right)=\overset{K-1}{\underset{k=0}{\sum }}v_{mk}Z_{k},\;m=1,\ldots ,M; \end{array}$$

that is, *F* = Φ**f**≃*V*_*p*_Λ**f**. *V*_*m*_ = (*v*_*m*1_,…, *v*_*mK*_) is the *m*th row of interpolation matrix *V*_*p*_ which makes use of *p* neighboring uniform samples of *Z* for approximation of each nonuniform sample of *F*. In [51], Fessler and Sutton designed a kind of min-max criterion to choose interpolation coefficients for every *F*(*ω*_*m*_):

(24) $$\begin{array}{c} \underset{V_{m}}{\min}\;\underset{{||f||}_{2}\leq 1}{\max}{\left|V_{m}\Lambda f-\phi_{m}f\right|}^{2}. \end{array}$$

The analytical solution of (24) is

(25) $$\begin{array}{c} V_{m}=\phi_{m}\Lambda^{H}{\left(\Lambda \Lambda^{H}\right)}^{-1}, \end{array}$$

where *H* denotes Hermitian transpose. Fessler and Sutton have shown that the overall complexity of such method is *O*(*qN*log⁡*N* + *pM*).

## 4. Simulation and Results

### 4.1. Simulation Parameters

**Figure 4:**
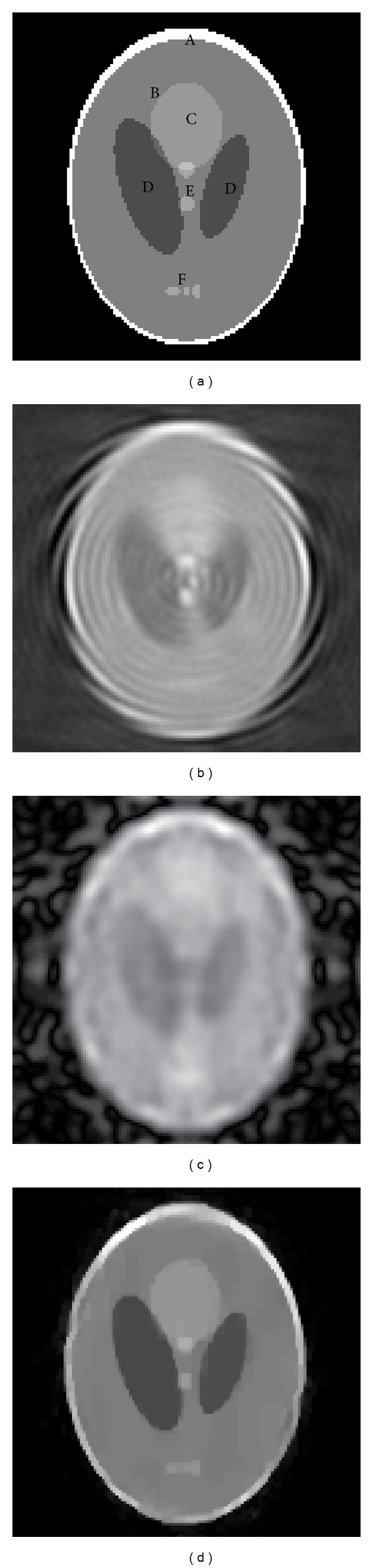
(a) Original image. (b) Image reconstructed using interpolation method. (c) Image reconstructed using broadband signal. (d) Image reconstructed using CS.

In order to evaluate the performance of the proposed method for UDT reconstruction, we have performed a series of numerical experiments for the phantom in Figure 4(a). The phantom consists of ten ellipses which looks like the well-known Shepp-Logan “head phantom” for CT imaging. However, for UDT system, we have modified the gray levels to those used by [8, 9]. The gray levels represent the relative change in refractive index from the background value of 1.0; the maximum and minimum gray intensity are set to 1.0 and 0, respectively. The speed of sound of the background media is 1500 m/s. To evaluate our method, the scattered field was calculated based on FDT under Born approximation. Although the Born approximation imposes limitation on the dimension of the object for real application [4] and cannot distinguish the features of the object spaced less than *λ*/2 [52, 53], it can provide a simple and direct method to reconstruct the structure of an object from the measurement of the scattered field. According to FDT, the Fourier transform of the scattered field measured on *η* = *l* is proportional to Fourier transform of the object over an arc (2), while the Fourier transform of each ellipse has simple analytical expression; hence we can generate the scattered data through inverse Fourier transform. This procedure not only is fast but also allows the scattered date to be calculated for testing the reconstruction algorithms and experiments parameters such as pitch and number of elements [4, 8–10].

In numerical experiments, the imaging system utilizes a pair of parallel linear array probes [54, 55]. Referring to exiting commercial ultrasound linear transducer and ultrasound tomography system, the frequency of incident wave is set to 1.5 MHz, and the number of elements and pitch of the probes are 128 and *λ*, respectively, where *λ* is the wavelength of incident plane wave. The distance between the two probes is 200*λ*. The phantom Figure 4(a) is discretized on a 128 × 128 Cartesian grid. According to the diffraction limitation [52, 53], the spatial sample step *T* or the resolution of the system is set to *λ*/2. It is also necessary to point out that the numbers of iterations for iterative methods are all set to 8; we did not employ error threshold as the iteration criterion, because we want to compare the iterative algorithms after the same iteration numbers. According to the recommendation of Fessler and Sutton [51], the values of *p*, *q* are set to 6 and 2, respectively. The regularization parameters *α* and *β* are set to 0.01 and 0.001, respectively.

### 4.2. Results

Figure 4 shows the reconstructed images through different methods from simulated sparse-view data with no added noise. Figure 4(a) is the original phantom, Figure 4(b) is the reconstructed image using bilinear frequency interpolation, Figure 4(c) is the reconstructed image using the method proposed by Bronstein et al. [10] with broadband incident sound wave, and Figure 4(d) is the reconstructed image using the proposed method where the sparse transform basis is Haar wavelet. The number of view is 16, and the number of iterations is 8 for Figures 4(c) and 4(d). Due to sparse-view sampling, the Nyquist-Shannon sampling limitation cannot be satisfied. The reconstructed image of Figure 4(b) is severely blurred and distorted by interpolation error. The small scale features of original phantom cannot be recognized and larger ones are distorted by ring artifacts. Compared with the interpolation method, the two iterative methods can suppress the artifacts and reduce the noises and Gibbs effect as shown in Figures 4(c) and 4(d). In Figure 4(c), we adopted ten different frequency waves used by Bronstein in [10] to illuminate the object in each view. The image shows oversmoothing effect that the edges and details of the features are blurred. This may be caused by resolving the inverse problem of UDT under overdetermined framework; that is, there are more known *F* than unknown *f*_*d*_.

Figure 4(d) shows the result of the proposed method. Most of the features can be clearly represented. The artifacts and oscillation noises are efficiently reduced. For the homogenous background (black region), there is no visible artifact and grayscale aberration, which accords with the characteristic of the original homogenous medium. For the narrow ring region *A*, the outer and inner boundary can be preserved. The inner background *B* is uniformly displayed except for parts of slightly blurred region. *C* is set to be a low-contrast region (the gray intensity is 0.5, while it is 0.6 for background *B*), which can be used to evaluate the sensitivity of imaging methods. In Figure 4(d), the region *C* can be clearly distinguished from the surrounding background with a sharp edge definition. Regions *D* and *E* are low and high refractive regions, with the gray intensity 0.3 and 0.75, respectively. The boundary of *D* is preserved and the inner of *D* is clean. The small region *E* can be distinguished from surroundings. It is worth noting that the three small ellipses at region *F* can be identified in Figure 4(d), while they are almost invisible in Figures 4(b) and 4(c). This implies the proposed method can improve the resolution efficiently in sparse-view situation.

**Figure 5:**
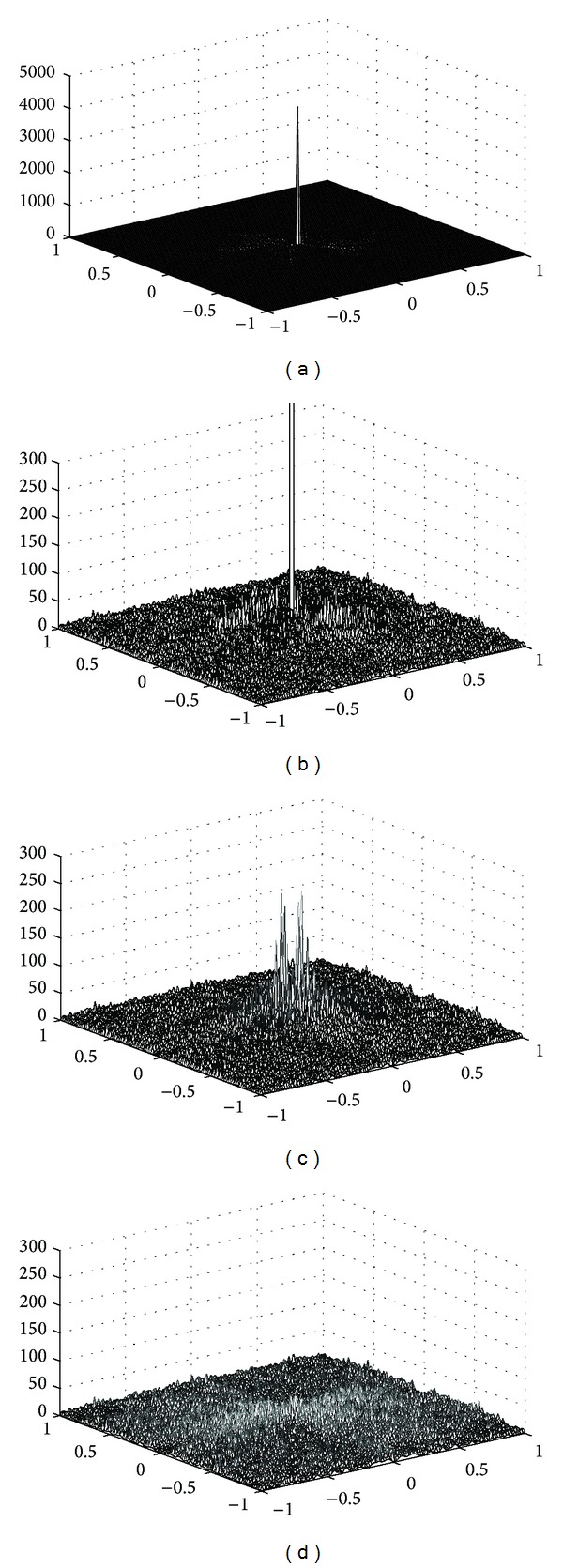
Magnitude of error in frequency domain. (a) Interpolation method; (b) error value of interpolation method within [0, 300]; (c) broadband method; (d) CS method.

Figure 5 shows the magnitude of error in the frequency domains. Since the maximum error of the interpolation method in Figure 5(a) is 4311, which is much bigger than the corresponding ones of the broadband method in Figure 5(c) (249.6) and the proposed method in Figure 5(d) (54.2), we also show the magnitude of error within the interval of [0,300] for the interpolation method (Figure 5(b)). From Figure 5, we can find that the error of interpolation method around center frequency is significantly larger than the remaining two methods, which accords with Figure 4(b) that the details of the features are seriously distorted comparing to other two methods. In Figure 5(c), the maximum error around center frequency is lower than that of Figure 5(b); however, the error of frequencies other than center frequency area is generally higher than the corresponding ones in Figure 5(b). This means the noises in the reconstructed image through broadband method are higher than the reconstructed image through interpolation method. Figure 5(d) shows that the proposed method can markedly reduce the frequency domain error particularly in the low frequency components. This indicates that the reconstructed image can faithfully represent the feature details while efficiently suppressing the noises for sparse-view sampling data.  Table 1 gives the relative mean square error (RMSE) in frequency domains, which is defined as

(26) $$\begin{array}{c} \text{RMSE}=\frac{{||F-\hat{F}||}_{2}^{2}}{{||F||}_{2}^{2}}; \end{array}$$

*F* and $\hat{F}$ are the distribution function of the original phantom and the reconstructed object in frequency domain, respectively.  Table 1 also lists the structural similarity (SSIM) index for different methods. Compared with RMSE, the SSIM has proven to be consistent with human eye perception [56]. In this paper, the original object (Figure 4(a)) is the reference image for SSIM. Compared to interpolation method, the proposed method has relatively smaller RMSE and higher SSIM values which coincides with the description above.

**Figure 6:**
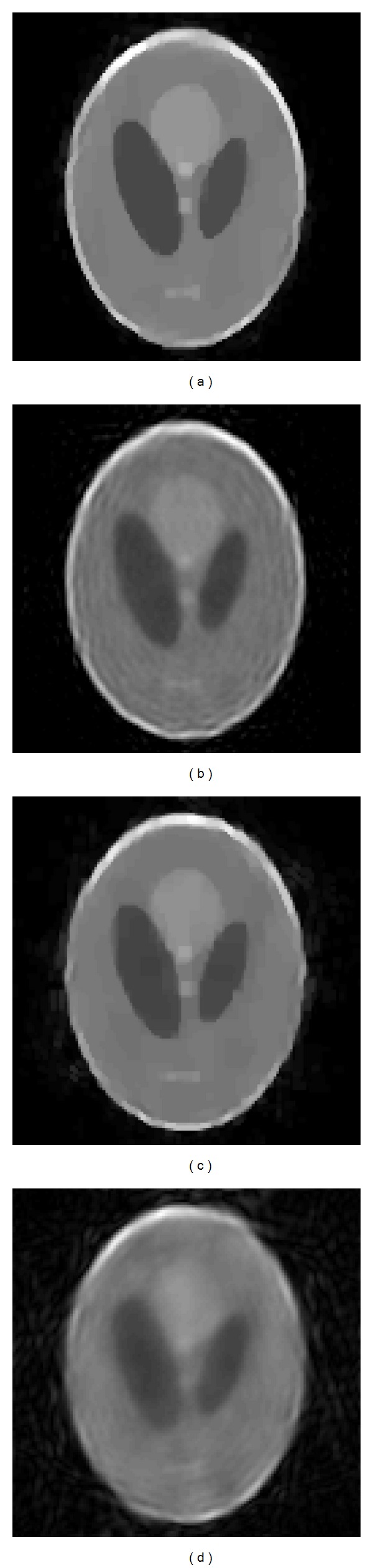
Images reconstructed with different penalties: (a) *α* = 0.01, *β* = 0.001; (b) *α* = 0.01, *β* = 0; (c) *α* = 0, *β* = 0.001; (d) *α* = *β* = 0.

In (15), the regularization parameters *α*, *β* determine the trade-off between the data consistency and the sparsity of the object. Furthermore, they can be used to adjust the relative weights of the different components in the cost function.  Figure 6 shows the images reconstructed under different penalties with 16 views, 128 elements, and pitch of *λ*.  Figure 6(a) is reconstructed with TV and *l*_1_ norm regularization terms the same as Figure 4(d); the regularization parameters are set to 0.01 and 0.001, respectively.  Figure 6(b) is reconstructed with solely TV regularization term; that is, *α* = 0.01, *β* = 0. Compared to Figure 6(a), although the TV penalty can preserve the edges of the object, the inner of the object is blurred.  Figure 6(c) is reconstructed with solely *l*_1_ norm regularization term; that is, *α* = 0, *β* = 0.001. The quality of the image, Figure 6(c), for inner region can match Figure 6(a), but the edges of the object are distorted.  Figure 6(d) is directly reconstructed from the limited sample data without any penalties; that is, *α* = *β* = 0. Compared to Figure 6(a), not only are the qualities of the inner and edge seriously declined in Figure 6(d), but also the background is filled with noise. In practical, one can choose the regularization parameters *α*, *β* empirically based on the noise level and image contrast [57] or adaptively adjust the regularization parameters in the process of reconstruction [58, 59]. The primary target of this work is to accurately reconstruct the object in sparse-view, the selection of the optimal regularization parameters is not the focus of this paper, but it is worth further investigation.

**Figure 7:**
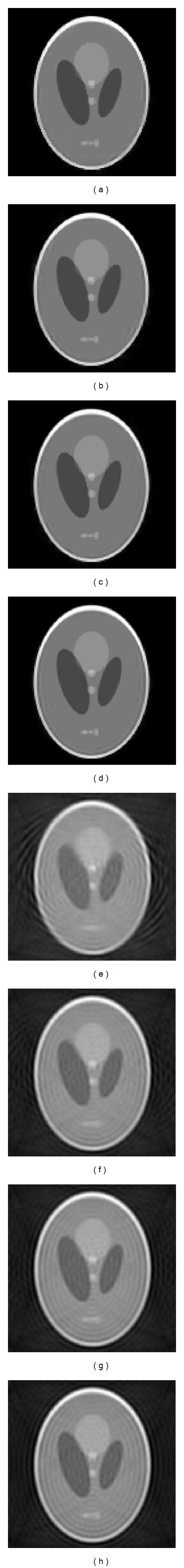
The reconstructed image through CS and interpolation methods with different views. (a), (b), (c), and (d) Reconstructed images by CS for 32 views, 48 views, 64 views, and 96 views, respectively; (e), (f), (g), and (h) reconstructed images by interpolation for 32 views, 48 views, 64 views, and 96 views, respectively.

To fully evaluate the proposed method, we also reconstruct the object with various numbers of views (Figure 7). In Figure 7(b), with 32 views, the smaller scale features (the three small ellipses) can be fully distinguished. Although the quality of reconstructed image for interpolation is also improved visually with the increase of views, the ring artifacts and oscillation noises cannot be eliminated even with 96 views.

## 5. Discussion

Not only can the sparse-view sampling scheme save the scan time of UDT but also it can reduce the complexity of the imaging device. In Section 4.2, we show the feasibility of image reconstruction for UDT in sparse-view situation based on CS framework. The two main reasons that CS is efficiently employed can be concluded as follows. Firstly, the acoustic indexes of the object are sparsely represented through orthogonal transform and finite difference transform; the former provides sparse representations for rapidly varied regions through wavelet transform, while the latter affords sparse transformation for piecewise smooth regions by TV. Secondly, the acoustic index coefficients with finite main components in the transform domain can be faithfully recovered through the iterative method based on CG and NUFFT.

To full evaluate our method, we will analyze the noise robustness of our method. Besides that, the computational complexity is also discussed in this part.

### 5.1. Robustness to Noise

In real situation, in addition to systematic errors such as misaligned transducers, the detected signals contain different kinds of noises such as thermal noise of transducer, electronic noise of amplifier. We added white Gauss noise in the received scatter data to test the robustness to noise of the proposed method. The signal-to-noise ratio (SNR) of simulated noisy data is 20 dB and 10 dB, respectively, where SNR is defined as

(27) $$\begin{array}{c} \text{SNR}=10\log\left(\frac{{||F||}_{2}^{2}}{{||n||}_{2}^{2}}\right). \end{array}$$

The pitch and the number of elements are *λ* and 192, respectively.

**Figure 8:**
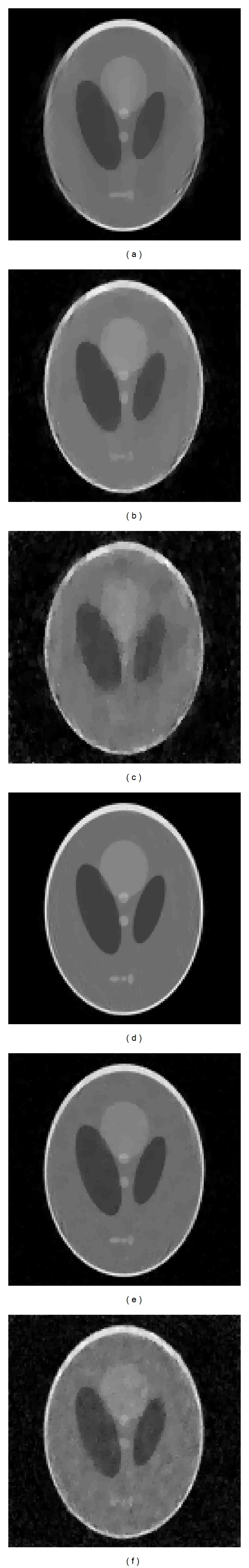
The robustness to noise of the proposed method. (a) 16 views, noise-free; (b) 16 views, SNR = 20 dB; (c) 16 views, SNR = 10 dB; (d) 32 views, noise-free; (e) 32 views, SNR = 20 dB; (f) 32 views, SNR = 10 dB.

Figure 8 shows the reconstructed images with none, 20 dB, and 10 dB noise under 16 views and 32 views. The images reconstructed from the signal with an SNR of 20 dB have hardly any difference with the images reconstructed from noise-free signals. The homogenous medium background is not contaminated by noise. The edges of different structures can be distinguished.

However, when the noise increases to 10 dB, the quality of reconstructed image is affected by granulation noise. Although the main features of the object are still visible, the small details are seen to seriously deteriorate, especially for 16 views. How to increase the robustness to noise remains a further research topic.

### 5.2. Computational Complexity

In the proposed method, the computation time is mainly occupied by NUFFT. For an *N* × *N* digital object, the complexity of NUFFT is *O*(2*qN* ^2^log⁡*N* + *pN* ^2^) [51], where *q* is the oversampling constant and *p* is the number of neighbors for interpolation. For normal reconstruction with the iteration number *m*_1_ and the linear search times *m*_2_ for each iteration, the total complexity of our method is estimated as *O*(2*m*_1_ *m*_2_(*qN* ^2^log⁡*N* + *pN* ^2^)). For comparison, the theoretical complexity of the frequency domain interpolation requires *O*(*N* ^2^log⁡*N* + 4*N* ^2^). Furthermore, the practical view number is generally more than 4*N* to avoid aliasing. The sample points for every view are at least two times of *N*. The complexity of filtered backpropagation (FBP) is about *O*(*N* ^3^log⁡*N*). For the 128 × 128 tested image used in this paper, the frequency interpolation method requires about 1.80 × 10^6^ operations; spatial domain interpolation method (FBP) would require 14.68 × 10^6^ operations and our method requires about 144.17 × 10^6^ operations.

## 6. Conclusion

UDT is an important image modality and can afford functional exams in application. CS is one of the most exciting advances in signal theory which takes advantage of compressibility of the object to break Nyquist limitation and recover the major component in transform domain. The paper presents one CS framework for UDT image reconstruction. The numerical experiments show that the proposed method can improve image quality. The relative error is smaller than conventional interpolation method and broadband method. Combining *l*_1_ norm with TV, not only is the edge of the object preserved, but also the contrast and resolution are improved.

In this paper our effort mainly focuses on integrating CS and UDT to develop practical framework for image reconstruction. Future work will be done on the evaluation of our study with in vivo data. Other important works include how to choose suitable transform basis and how to choose regularization parameters.

## Figures and Tables

**Table 1:** SSIM and RMSE for different methods.

	Interpolation	Broadband signal	CS
SSIM	0.291	0.346	0.820
RMSE in frequency domain	0.765	0.352	0.255
